# Ashwagandha-Induced Hepatic Injury: A Case Report

**DOI:** 10.7759/cureus.71576

**Published:** 2024-10-15

**Authors:** Fakhruddin Almuzghi, Abubaker Abdelmalik, Maaly Abuhlaiga

**Affiliations:** 1 Department of Internal Medicine, Hamad Medical Corporation, Doha, QAT; 2 Faculty of Medicine, Misurata University, Misrata, LBY; 3 Division of Nephrology, Department of Internal Medicine, Misrata Medical Center, Misrata, LBY

**Keywords:** ashwagandha (withania somnifera), clinical case report, drug-induced liver injury (dili), jaundice, libya

## Abstract

A 22-year-old healthy Libyan female suffered moderate to severe liver injury following the ingestion of ashwagandha capsules. Following a latency period of 30 hours, she developed severe itching, fatigue, nausea, and jaundice. Laboratory results showed significantly increased liver enzymes (especially alanine aminotransferase (ALT)) and bilirubin (mainly conjugated bilirubin), along with a slight elevation of alkaline phosphatase (ALP). We used the Roussel Uclaf Causality Assessment Method (RUCAM) worksheet to link liver injury to ashwagandha. The R ratio was 5.4, and the overall RUCAM score was seven. The peaks of ALT, total bilirubin, and ALP were 315 IU/L, 12.85 mg/dl, and 150 IU/L, respectively. The washout period was 60 days. Symptoms were relieved by ursodeoxycholic acid (UDSA). The clinical assessment and workup for viral hepatitis and autoimmune hepatitis were negative. In conclusion, ashwagandha can cause prolonged hepatocellular liver injury in healthy and young individuals.

## Introduction

Drug-induced liver injury (DILI) is a common cause of liver injury. Diagnosing DILI is challenging because there is no specific sign or test to confirm the diagnosis. Generally, DILI is considered a diagnosis of exclusion in clinical practice. Drug-induced liver injury is caused by many medications (prescribed or over-the-counter) and even herbal and dietary supplements. The clinical presentation of DILI is similar to liver injury secondary to infectious or malignant diseases. Therefore, a high index of suspicion is required to diagnose it. According to the degree of alkaline phosphatase (ALP) and alanine aminotransferase (ALT) elevation, it is classified into hepatocellular, cholestatic, and mixed liver injury. Ashwagandha (*Withania somnifera*), known for its ayurvedic use, has recently garnered much attention worldwide in modern practice remarkably for its anxiolytic effect. However, the data supplemented surrounding its use and side effect profile are quite limited, in particular, adverse reactions pertaining to the hepatobiliary system. Upon searching on PubMed, one will find a single five-patient case series [1]. According to the LiverTox database, ashwagandha is classified as a probable cause of clinically apparent liver injury or formally a likelihood score of C [2]. 

## Case presentation

Patient history

A 22-year-old female Libyan medical student presented to the clinic complaining of intense generalized itching. Symptoms started 30 hours following the ingestion of ashwagandha capsules. Incentivized by a social media advertisement, the patient opted for the drug as a means to relieve medical exam anxiety. She endorsed a consumption of three capsules (450 mg/capsule) eight hours apart on the first day, followed by two more on the second day. This was the recommended dose by the manufacturer, and she denied overdose intake. Approximately 30 hours beyond the first intake, symptoms emerged. On the day of presentation at the clinic, she reported itching, malaise, anorexia, and nausea for seven days. Symptoms were progressive and unremitting, with itching interfering with daily activities, dark urine for two days, and jaundice for one day. Skin scuffing could not relieve the itching, nor was it altered by hot or cold showers. It was not associated with any other gastrointestinal symptoms, rash, or flu-like symptoms. The patient reported no history of similar complaints. There was no history of sick contact or recent travel. She was otherwise healthy, was not taking any medication, and had no allergies. Her family medical history was unremarkable. No history of smoking, alcohol consumption, or illicit drug use was noted.

Physical examination

On examination, she was vitally stable, appeared to be in discomfort, and was constantly itching and fidgety. Scleral icterus was visible, and skin excoriation marks were present on all limbs. Regarding other stigmata of liver disease, no palmar erythema, asterixis, bruises, abdominal distention, organomegaly, lower extremity edema, spider naevi, digital clubbing, or lymphadenopathy were noted. No allergic signs, such as hives, stridor, wheezing, or facial edema, were evident. There was no evidence of arthritis, mucosal lesions, or needle track marks. Examination of the respiratory, cardiovascular, and nervous systems was normal.

Laboratory results and imaging

All initial investigations, except for the liver function test, were within normal limits (Table 1). The blood test was taken at presentation (i.e., seven days post symptom onset). Transaminases were elevated with an aspartate aminotransferase (AST)/ALT ratio of less than 1, and the R ratio was 5.4. Total bilirubin was 2.63 mg/dL, with direct being 2.53 mg/dL. Albumin and prothrombin time (PT) were, however, normal. The hepatitis panel was also negative for viral hepatitis. The extractable nuclear antigen (ENA) panel was also negative (Table 2). In addition to the laboratory workup, an ultrasound of the abdomen and pelvis was performed by an expert radiologist, demonstrating no abnormal pathology. 

**Table 1:** Initial blood test results WBC: white blood cells; RBC: red blood cells; Hb: hemoglobin; MCV: mean corpuscular volume; HTC: hematocrit; HCHC: mean corpuscular hemoglobin concentration; PLT: platelet; Na: sodium; K: potassium; Cl: chloride; HCO3: bicarbonate; Cr: creatinine; ESR: erythrocyte sedimentation rate; CRP: C-reactive protein; ALP: alkaline phosphatase; AST: aspartate transaminase; ALT: alanine transaminase; GGT: gamma-glutamyl transpeptidase; PT: prothrombin time; INR: international normalized ratio; aPTT: activated partial thromboplastin time; TSH: thyroid-stimulating hormone

Initial blood test results		
	Patient results	Normal range
WBC	5.34 x 10*9/L	4 - 10
Lymphocytes	27%	-
Neutrophils %	59%	-
Monocytes %	10%	-
Eosinophils %	4%	-
Basophils %	0%	-
RBC	4.66x1*12/L	3.8 – 4.8
Hb	13.1 g/dL	12 - 15
MCV	79.6 f/L	83 - 101
HCT	37 %	36 - 46
MCH	28 pg	27 - 31
MCHC	35.4 g/dL	32 - 36
PLT	348x10*9/L	150 - 410
Blood group	O, Rh-negative DU negative	-
Na	138 mEq/L	133 - 146
K	3.4 mEq/L	3.5 - 5.3
Cl	101 mEq/L	95 – 108
HCO3	27 mEq/L	22 - 29
Urea	20 mg/dl	5 - 20
Cr	0.5 mg/dl	0.6 - 1.3
ESR	15-38 mm/2hou	< 20 mm/hr
CRP	7.1 mg/l	0 - 5
Amylase	70 U/L	40 - 140
ALP	118.8 IU/L	44 - 147
GGT	17 U/L	5 – 40
AST	104 IU/L	8 – 33
ALT	185.7 IU/L	4 – 36
Total bilirubin	2.63 mg/dl	0.1 - 1.2
Direct bilirubin	2.53 mg/dl	< 0.3
Indirect bilirubin	0.1 mg/dl	-
PT	13.4 seconds	12.3 - 15.1
INR	1	-
aPTT	32 seconds	29.0 - 43.0
Total protein	87 g/L	60 - 85
Uric acid	3.7 mg/dl	3.5 - 7.2
Ferritin	145.17 ng/ml	13 - 150
Triglyceride	158.9 mg/dl	< 150
Cholesterol	203.9 mg/dl	< 200
TSH	0.96 mIU/L	0.5 - 5.0
Free T4	14.14 pmol/L	10.3 - 24.5

**Table 2:** Extractable nuclear antigen (ENA) panel

Antibody	Result
Anti-HBc (Total)	Negative
ANTI-HBc (IgM)	Negative
Anti-HCV	Negative
Anti-HAV (IgM)	Negative
HBsAg	Negative
Anti dsDNA (AC-1) (dsDNA)	Negative
Anti nucleosomes (AC-1) (NUC)	Negative
Anti histones (AC-1) (HI)	Negative
Anti SS-A (AC-4) (SSA)	Negative
Anti Ro-52 (Ro-52)	Negative
Anti SS-B (AC-4) (SSB)	Negative
Anti RNP/Sm (AC-5) (RNP/Sm)	Negative
Anti Sm (AC-5) (Sm)	Negative
Anti Mi-2alpha (AC-4) (Mi-2a)	Negative
Anti Mi-2beta (AC-4) (Mi-2b)	Negative
Anti Ku (AC-4) (Ku)	Negative
Anti Centromere A (AC-3) (CA)	Negative
Anti Centromere B (AC-3) (CB)	Negative
Anti Sp100 (AC-6) (Sp100)	Negative
Anti PML (AC-6) (PML)	Negative
Anti Scl-70 (AC-8) (Scl-70)	Negative
Anti PM-Scl100 (AC-8) (PM100)	Negative
Anti PM-Scl75 (AC-8) (PM75)	Negative
Anti RP11 (AC-10) (RP11)	Negative
Anti RP155 (AC-10) (RP155)	Negative
Anti gp210 (AC-11) (gp210)	Negative
Anti PCNA (AC-13) (PCNA)	Negative
Anti DFS70 (AC-2) (DFS70)	Negative
Anti AMA-M2 (M2)	Negative
Anti LKM-1 (LKM)	Negative
Anti LC-1 (LC1)	Negative
Anti SLA/LP (SLA)	Negative
Anti SMA (SMA)	Negative

Management and follow-up

**Figure 1:**
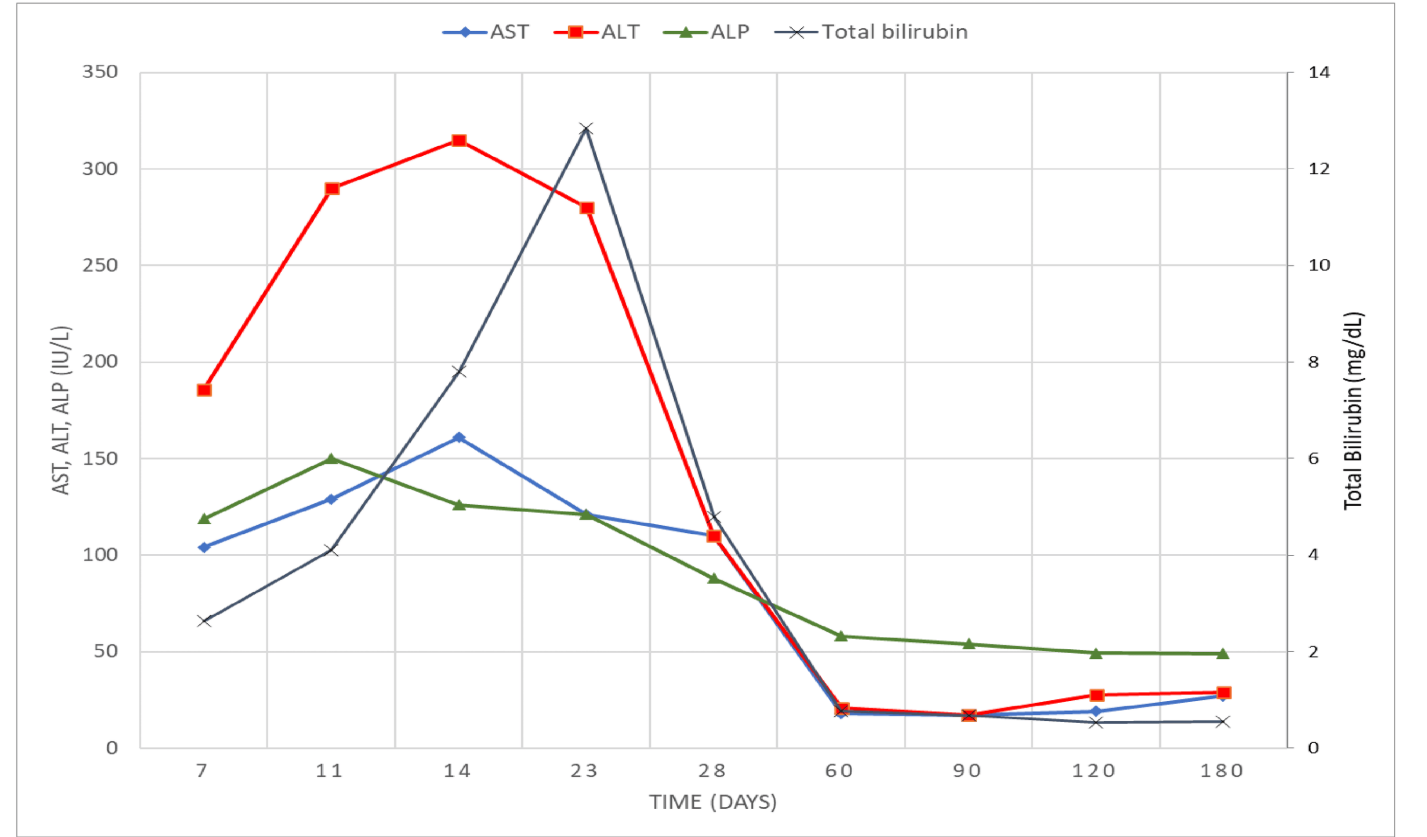
The trend of the liver function test during the follow-up period AST: aspartate transaminase; ALT: alanine transaminase; ALP: alkaline phosphatase

The patient was followed up clinically for six months either via clinic or a phone call. With interim blood tests during clinic visits to monitor the changes in the initially abnormal liver panel values and for reaffirmation of hepatitis serology. Management of the patient was focused mainly on cessation of the inciting drug and avoidance of any hepatotoxic supplements, along with symptomatic relief. Although the patients developed symptoms 30 hours after drug intake, the first laboratory abnormality was detected on day seven post ingestion. The time taken for the ALT and total bilirubin levels to peak was 14 and 23 days post ingestion, respectively (Figure 1). The R ratio at the peak of injury was 8.6. An antihistamine (desloratadine 5 mg) tablet once daily was prescribed for pruritus, but after three days stopped per the patient due to lack of relief. Then, cholestyramine 4 g sachets daily were supplied; however, it was immediately ceased on day 2 by the patient for symptom exacerbation. Thereafter, we prescribed ursodeoxycholic acid (USDA) capsules, 250 mg twice daily (10 mg/kg per day), which proved helpful in itch relief followed by a decrease in yellowish discoloration. The patient continued UDSA for 10 days. As far as liver function was concerned, it took approximately nine weeks to normalize (Figure 1). At the end of the follow-up, the patient did not have any complaints and physically had a resolution of jaundice. Repeat hepatitis panel, hepatitis B virus, and hepatitis C virus were later also negative. 

## Discussion

The patient had to be worked up extensively to exclude other causes of liver injury using the American College of Gastroenterology algorithm [3]. Moreover, the Roussell-Uclaf Causality Assessment Method (RUCAM) score can be used to pinpoint ashwagandha as the most probable culprit [2]. Furthermore, the type of liver injury can be classified as hepatocellular, cholestatic, or mixed using RUCAM. Our patient's R ratio was 5.4 at the presentation and peaked later at 8.6 during the follow-up period, which indicates hepatocellular liver injury. This differs from the only published case series from Iceland and the US, as they reported cholestatic liver injury in their cohort [1]. This is due to the mild elevation of ALP levels and a significant increase in liver enzymes observed in our patient. 

The RUCAM total score is computed based on eight factors from seven different categories (time to onset, course, risk factors, concomitant drugs, non-drug causes of liver injury, previous information on the hepatotoxicity of the drug, and response to rechallenge) which will help define the likelihood of the implicated medication being a culprit. The total score may range from -9 to +14; some categories could be excluded, such as response to rechallenge, which is rarely done (as in our case), making the range -7 to +11. Also, without risk factors, the score would encompass -7 to +9. Our patient score was seven out of nine, labeling the causality as "probable.”

Other common causality assessment tools for DILI include the Maria & Victorino system and the Clinical and Diagnostic Scale (CDS). Among many other tools, the RUCAM remains widely adopted as it performed moderately well in reproducibility and intraobserver variability, given its objective measurements. Another assessment tool we applied was the Drug-Induced Liver Injury Network (DILIN) scale to quantify the severity of the injury using a five-point scale [2]. Our patient had a grade 3 injury severity (i.e., moderate to severe) according to the DILIN scale. 

Our patient suffered gravely from this liver injury despite being young, effectively healthy, and free of potential hepatotoxic risk factors. Our concern is the fact that neither pathognomonic clinical findings nor diagnostic serologic markers make the diagnosis of DILI challenging. Coupled with its self-limiting course, we fear that it might be underreported and underdiagnosed. Therefore, individuals susceptible to hepatoxicity, such as the elderly with liver disease, should avoid its consumption until further research and understanding is acquired.

## Conclusions

Ashwagandha may cause moderate to severe liver injury to healthy young individuals. Concerning symptoms implicated include itching and jaundice, lasting up to nine weeks. Total bilirubin and ALT levels at the peak of the injury were 12 mg/dL and 315 IU/L, respectively. Liver injury can be hepatocellular, not just cholestatic. The washout period for this patient was two months. Neither antihistamines nor cholestyramine alleviates symptoms, but USDA can relieve symptoms.
